# Fully automated accurate patient positioning in computed tomography using anterior–posterior localizer images and a deep neural network: a dual-center study

**DOI:** 10.1007/s00330-023-09424-3

**Published:** 2023-01-27

**Authors:** Yazdan Salimi, Isaac Shiri, Azadeh Akavanallaf, Zahra Mansouri, Hossein Arabi, Habib Zaidi

**Affiliations:** 1 Division of Nuclear Medicine and Molecular Imaging, Geneva University Hospital, CH-1211 Geneva, Switzerland; 2 Geneva University Neurocenter, Geneva University, Geneva, Switzerland; 3 Department of Nuclear Medicine and Molecular Imaging, University of Groningen, University Medical Center Groningen, Groningen, The Netherlands; 4 Department of Nuclear Medicine, University of Southern Denmark, Odense, Denmark

**Keywords:** Computed tomography, Patient positioning, Deep learning, CT localizer

## Abstract

**Objectives:**

This study aimed to improve patient positioning accuracy by relying on a CT localizer and a deep neural network to optimize image quality and radiation dose.

**Methods:**

We included 5754 chest CT axial and anterior–posterior (AP) images from two different centers, C1 and C2. After pre-processing, images were split into training (80%) and test (20%) datasets. A deep neural network was trained to generate 3D axial images from the AP localizer. The geometric centerlines of patient bodies were indicated by creating a bounding box on the predicted images. The distance between the body centerline, estimated by the deep learning model and ground truth (BCAP), was compared with patient mis-centering during manual positioning (BCMP). We evaluated the performance of our model in terms of distance between the lung centerline estimated by the deep learning model and the ground truth (LCAP).

**Results:**

The error in terms of BCAP was − 0.75 ± 7.73 mm and 2.06 ± 10.61 mm for C1 and C2, respectively. This error was significantly lower than BCMP, which achieved an error of 9.35 ± 14.94 and 13.98 ± 14.5 mm for C1 and C2, respectively. The absolute BCAP was 5.7 ± 5.26 and 8.26 ± 6.96 mm for C1 and C2, respectively. The LCAP metric was 1.56 ± 10.8 and −0.27 ± 16.29 mm for C1 and C2, respectively. The error in terms of BCAP and LCAP was higher for larger patients (*p* value < 0.01).

**Conclusion:**

The accuracy of the proposed method was comparable to available alternative methods, carrying the advantage of being free from errors related to objects blocking the camera visibility.

**Key Points:**

• *Patient mis-centering in the anterior–posterior direction (AP) is a common problem in clinical practice which can degrade image quality and increase patient radiation dose*.

• *We proposed a deep neural network for automatic patient positioning using only the CT image localizer, achieving a performance comparable to alternative techniques, such as the external 3D visual camera*.

• *The advantage of the proposed method is that it is free from errors related to objects blocking the camera visibility and that it could be implemented on imaging consoles as a patient positioning support tool*.

## Introduction

Computed tomography is a valuable imaging modality in the diagnosis of a wide variety of pathologies. Optimizing scanning parameters, patient positioning, and scan range are critical to maximize the diagnostic value and minimize the radiation risks to patients. A number of studies reported on debatable practices in clinical setting resulting from inadequate selection of scan-related factors, such as patient positioning, scan range selection, irradiation parameters, and reconstruction methods. It is manifest that these imprecisions in parameter selection can have more negative effects when automatic methods, such as tube current modulation (TCM) and automatic kVp selection, are implemented [1].

In a recent study, Akintayo et al [2] reported a high prevalence (more than 80%) of patient mis-centering in the *Y*-axis (table height) after evaluating a large cohort consisting of 57,621 CT scans. An average mis-centering error of 14.7 ± 17 mm was reported in chest CT scanning. Sukupova et al [3] demonstrated the high prevalence (470 from 473 cases) of mis-centering in clinical practice with an average mis-centering of  −43 mm. Differences between patients’ body centerline and the gantry isocenter would lead to additional doses and degraded image quality. Li et al [4] reported up to 30% surface dose increment due to 60 mm mis-centering in a cylindrical water phantom. Furukawa et al [5] evaluated the effect of table height on the behavior of the TCM system. They showed that a higher table height causes a magnification in the anterior–posterior (AP) localizer, consequently overestimating the attenuation and increasing the radiation flux through tube current elevation.

Euler et al [6] evaluated the effect of table mis-centering from  −60 mm to  +60 mm on organ doses measured on a 5 y/o anthropomorphic phantom using semiconductor dosimeters. They reported up to 28% change in organ doses in chest scanning range. In their first study, Kaasalainen et al [7] showed more than 100% change in organ doses (such as the thyroid and lungs) measured in an anthropomorphic phantom due to patient mis-centering. In their second study, Kaasalainen et al [8] reported up to 91% change in the CTDI_vol_ by changing the table height. A number of studies attempted to overcome the mis-centering issue through automatic patient positioning, mainly employing an additional visual camera for 3D imaging. In serial studies, Booij et al [9, 10] calibrated a 3D camera fixed on the ceiling to position the patient automatically and tested the performance of their technique on 254 adults and 191 pediatric patients. The median error of their method was 5.4 mm after excluding outliers in adult cases. Moreover, after excluding outliers, they obtained an error of 4.8 mm on 191 pediatric patients. Dane et al [11] reported an error of 6.8 ± 6.1 mm utilizing a 3D camera, an AP localizer, and human intervention.

Gang et al [12] scanned 127 patients twice with manual and automatic positioning. Their results showed a 15.6 ± 8.3 mm error from the patients’ centerline, wherein mis-centering correction improved image quality in terms of noise and lesion signal-to-noise ratio (SNR) while decreasing the radiation dose. Saltybaeva et al [13] reduced manual positioning errors for chest CT images from 19 to 7 mm using a 3D camera. A 3D camera installed in the scanning room is not always available and commonly requires sensitive/precise calibration procedures. Moreover, a portable camera is also prone to numerous errors [14]. Besides, using a camera would lead to considerable mis-centering errors, much more than manual setups, in cases where additional/extra objects are located on the patient’s body, such as a blanket or respiratory aiding or tracking device [15]. This would significantly limit the application of this method in routine clinical practice.

Deep learning (DL) has demonstrated excellent performance in automating multiple medical image analysis tasks, including segmentation [16–20], computational modeling [21, 22], radiation dosimetry [23, 24], scan range selection [25], low-dose imaging [26–28], and protocol optimization [29]. The use of DL to automate patient positioning in CT scanning is very sparse, with only a few of studies so far [30]. In this context, we explored the possibility of automatic patient-specific positioning for CT examinations. The main purpose of the current study was to automate the detection of the patient’s body centerline distance from the gantry isocenter with the aim to perform automatic patient positioning in chest CT scans by means of deep learning algorithms using only the AP localizer as input.

## Material and methods

### Study population

**Fig. 1:**
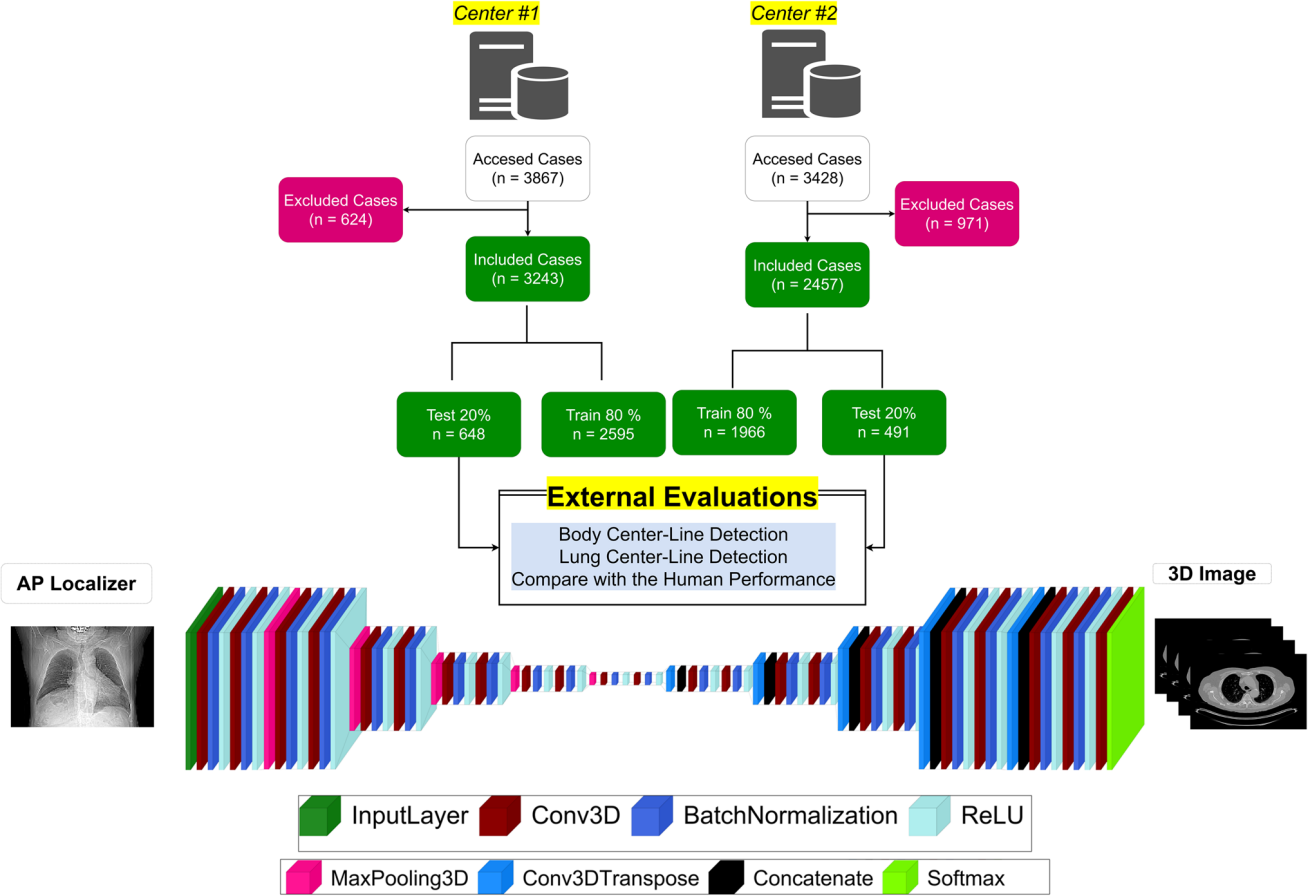
Flowchart of the overall process implemented in this study protocol along with and the architecture of the employed U-NET deep neural network

We collected 7295 chest CT images acquired from two imaging centers equipped with Siemens Somatom Duo (C1, 3867 cases, 2066 male and 1801 female) and Phillips Brilliance 16 (C2, 3428 cases, 1728 male and 1700 female). The patients were referred for the assessment of different pathologies in the thorax region, where chest CT imaging was requested, excluding cardiac and spine indications. Our study was approved by the local ethics committees, and the written informed consent was waived owing to the retrospective nature of the study. The 2D AP localizer and 3D axial images were collected in DICOM format. We excluded 624 and 917 cases from C1 and C2 databases, respectively. The main criteria for exclusion were truncation artifacts because indicating the anterior, posterior, and consequently, the AP centerline borders is not possible from a truncated image. The remaining 5754 cases (3243 cases from C1 and 2511 cases from C2 centers) were included in the study protocol (Fig. 1).

### Single-slice reconstruction

#### Image pre-processing

The localizer and axial 3D images were cropped to the same dimension ratio using the location tag stored in the DICOM headers. The localizer images were normalized to [0–1] intensity range and resized to a matrix size of 144 (lateral direction) by 64 (Z-direction) pixels. The axial images were reoriented to coronal orientation and then resized to a matrix of 184 (AP direction), 144 (lateral direction), and 64 (Z-direction) voxels, followed by intensity normalization to the range [0–1].

#### Neural network architecture and training

The data were randomly split into training (80%) and test (20%) datasets for each center. It should be noted that, inspired by a previous study, which demonstrated that the localizer pixel values and geometrical characteristics of the scout view images are scanner-specific [25], we trained two separate models using data from each scanner. A modified U-NET deep neural network was used with five encoder and five decoder layers using Matlab-based deep learning module (Fig. 1). Each encoder and decoder contained a batch normalization and ReLU with stride of 2 × 2. The input was the pre-processed AP 2D localizer images, whereas the output was the pre-processed 3D coronal image. The training was performed on a PC equipped with an NVIDIA 2080 TI GPU with 11 GB of RAM. The training was continued for 50 epochs with a batch size of 1 and Adam optimizer. We did not use any data augmentation strategy.

### Centerline measurement

All ground truth and deep learning reconstructed images from the external group were reoriented in the axial orientation. The body contour was automatically extracted by applying an intensity-based thresholding and region shape evaluation by removing the table and other objects in the field of view, such as respiratory aiding tools, patient clothes, and blankets. A bounding box (B.Box) was created around the body, wherein the anterior body limit, posterior body limit, and the AP centerline of the body were calculated from the B.Box information. The body centerline extracted from deep learning reconstructed images (BCDL) was compared to the body centerline extracted from axial ground truth images (BCGTH). The deep learning–based automatic positioning error in the body center (BCAP) was defined as BCAP = BCDL − BCGTH (mm). The BCAP metric was considered as the ability of our algorithm to identify the centerline of the body from AP localizers, consequently indicating the distance of the body centerline from the gantry isocenter, which is a fixed location for any CT scanner, and finally, the ability in the automatic setting of the desired table height and patient positioning.

Similarly, the distance between BCGTH and the gantry isocenter was considered as manual positioning error according to the body center (BCMP) formulated as BCMP = BCGTH − gantry isocenter. BCMP was considered as metric for evaluating technologists’ accuracy in patient positioning according to body centerline.

Besides, the lungs were segmented by applying intensity-based thresholding on the axial ground truth images and the images generated by the deep learning model. Similar to the procedure followed for body contour delineation, the centerline for both lungs (LC) was calculated by creating a B.Box on both lung segments covering the whole lung. The centerline of the lung extracted from ground truth axial slices (LCGTH) and the centerline of the lung extracted from images generated by the deep learning model (LCDL) were separately measured. The error in deep learning automatic positioning according to the lung centerline was defined as LCAP = LCDL − LCGTH. The error of technologists in positioning the lung centerline in the isocenter was defined as LCMP = LCGTH − gantry isocenter.

The negative values for BCAP, LCAP, LCMP, and BCMP indicate patient positioning (centerline) under the gantry center (lower table height), whereas positive values indicate patient positioning above the gantry center (higher table height). For all cases included in this study (3243 cases for C1 and 2511 for C2), the BCMP and LCMP were measured to evaluate technologists’ performance in positioning the patients in the AP direction. We compared the DL and technologists’ performance for the external test groups (C1: 648 cases and C2: 502 cases).

### Statistical analysis

The ability of our algorithm to detect body centerlines was compared with the ground truth data obtained from 3D axial images. The Kolmogorov–Smirnov test was used to test the normality of the distribution. The BCAP and LCAP were compared with BCMP and LCMP, respectively, through the Mann–Whitney test. Moreover, we repeated the Man-Whitney test with the absolute values of BCAP, BCMP, LCAP, and LCMP as input to eliminate the counteracting effect of negative and positive errors. We used the Spearman test to find any potential correlation between body size and positioning errors. *p* values less than 0.05 were considered statistically significant.

## Results

### Population and scan parameter description

Table 1 summarizes the patients’ demographic information included in this study. The patients’ body sizes for center #2 (C2) were slightly larger than those of center #1 (C1), but this difference was not statistically significant (*p* value > 0.05).

**Table 1:** Patient demographics and CT acquisition parameters for the two centers included in the study protocol. *D_AP*, AP diameter; *D_Lat*, lateral diameter

Database	Male	Female	Age	D_AP (cm)	D_Lat (cm)	Scanner	kVp	Tube current (mA)	CTDI_vol_ (mGy)
C1	1731	1512	53.8 ± 17.9	24.3 ± 3.6	30.1 ± 5.3	Siemens	110	170.0 ± 38.5	6.21 ± 1.48
C2	1339	1172	47.4 ± 18.4	24.4 ± 2.9	32.1 ± 4.7	Phillips	90 and 120	92.45 ± 47.92	5.07 ± 3.41

The average errors, i.e., the absolute BCMP with respect to positioning for all 5754 cases included (C1: 3243 and C2: 2511 cases), were $14.50\pm 10.40$ and $16.55\pm 9.72$ mm, respectively. The absolute LCMP was $13.06\pm 14.4$ and $12.5\pm 9.81$ for C1 and C2 datasets, respectively. Body mis-centering of more than 10 mm occurred in 60% of the cases.

### Body and lung centerline detection

Table 2 shows the performance of our proposed DL-based method vs. human performance in clinical scenario. The error in automatic body centering in terms of BCAP was $-0.75\pm 7.73$ and $2.06\pm 10.61$ mm for C1 and C2, respectively, which is considerably better than the technologists’ performance in terms of BCMP for both centers of C1 and C2 (*p* value < 0.01). At the same time, the error in lung centering in terms of LCAP was $1.56\pm 10.8$ and $-0.27\pm 16.29$ mm for C1 and C2, respectively. The LCAP was significantly better than LCMP for the C1 dataset (*p* value  < 0.01), while no statistically significant difference was found between LCAP and LCMP for C2 test dataset (*p* value = 0.069). The total number (45 cases) had an absolute BCAP of more than 20 mm.

**Table 2:** Comparison of automatic positioning errors between automatic and manual positioning for the two centers. The *p* value shows the results of the Mann–Whitney statistical test

			Error (mm)			Absolute error (mm)		
			Mean ± SD	95% CI	*p* value	Mean ± SD	95% CI	*p* value
C1	Body	Human (BCMP)	9.35 ± 14.94	8.16–10.54	< 0.01	14.03 ± 10.66	13.18–14.87	< 0.01
		Deep (BCAP)	− 0.75 ± 7.73	− 1.22 to 0.14		5.7 ± 5.26	5.28–6.12	
	Lung	Human (LCMP)	8.53 ± 17.47	7.15–9.92	< 0.01	13.06 ± 14.4	11.92–14.21	< 0.01
		Deep (LCAP)	1.56 ± 10.8	0.7–2.42		7.49 ± 7.93	6.86–8.12	
C2	Body	Human (BCMP)	13.98 ± 14.5	12.7–15.25	< 0.01	16.08 ± 12.12	15.01–17.14	< 0.01
		Deep (BCAP)	2.06 ± 10.61	1.13–3.00		8.26 ± 6.96	7.65–8.87	
	Lung	Human (LCMP)	4.88 ± 15.13	3.54–6.21	< 0.01	12.5 ± 9.81	11.64–13.36	0.069
		Deep (LCAP)	− 0.27 ± 16.29	− 2.86		12.25 ± 10.72	11.3–13.19	

**Fig. 2:**
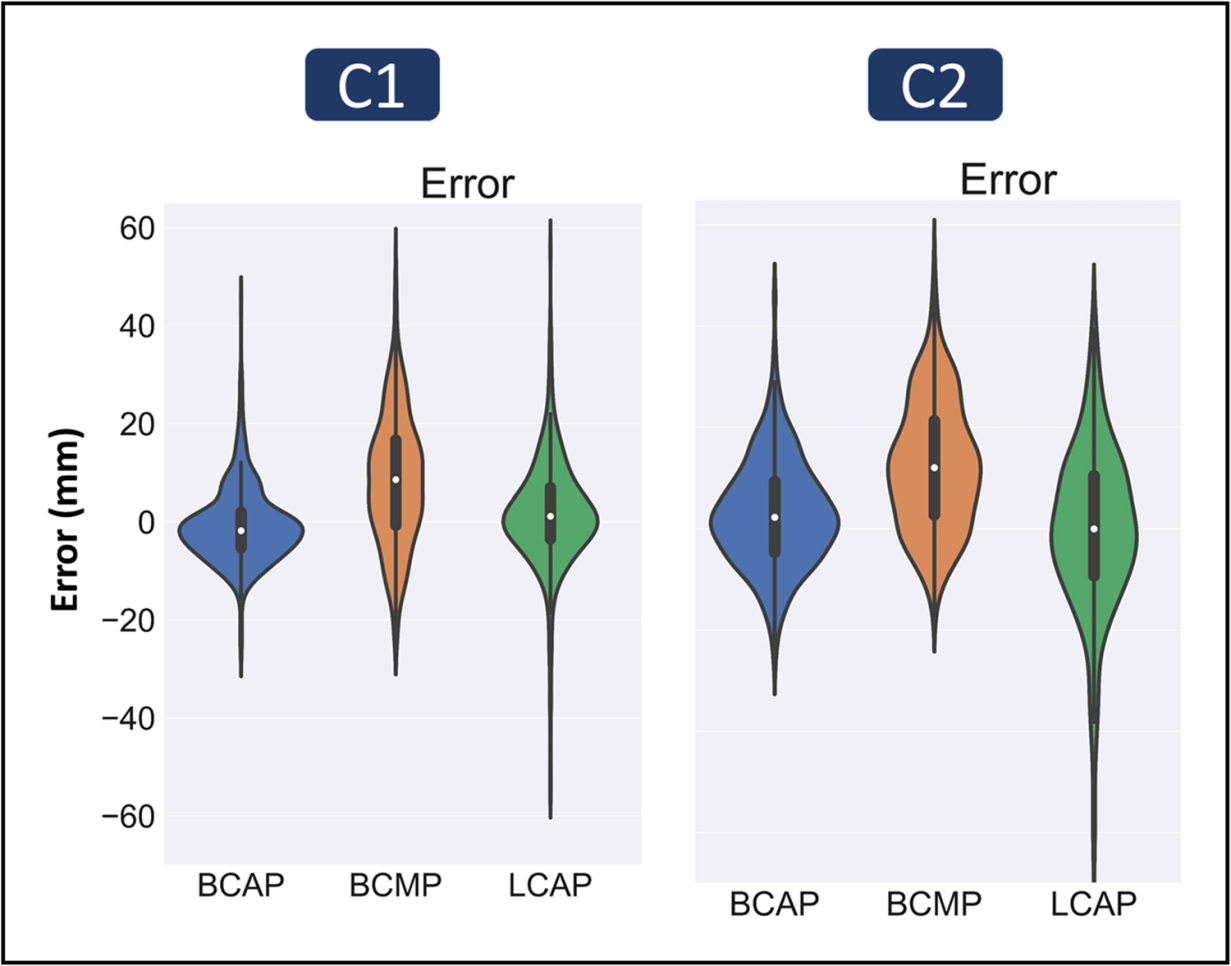
Comparison between the centerline detection error through DL and human performance

Figure 2 depicts violin plots comparing the DL-based approach and human performance for both centers with respect to body centering. The DL-based method exhibited superior performance in body centerline detection compared to lung centerline, with a lower bias and less variance (Table 2).

**Fig. 3:**
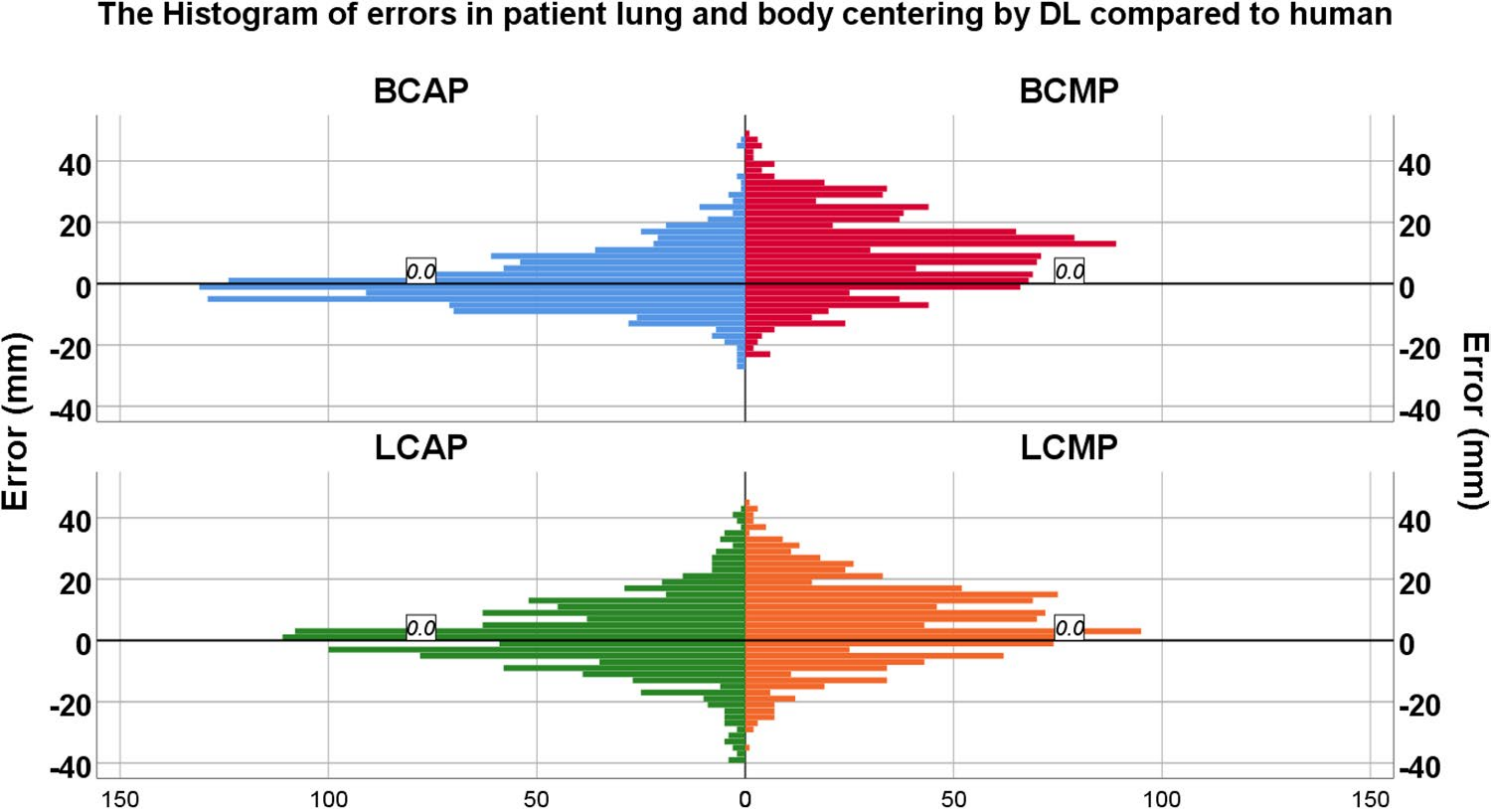
Histogram of errors in patient positioning achieved by manual and automatic positioning in terms of lung positioning errors of lung mis-centering during manual positioning (LCMP) and lung mis-centering during automatic positioning (LCAP) and body positioning errors of body mis-centering during automatic positioning (BCAP) and patient mis-centering during manual positioning (BCMP)

Figure 3 presents the distribution of errors from the gantry isocenter achieved by the automatic and manual positioning techniques. The DL-based method increased the accuracy of patient body centering, where the average absolute values of BCAP and BCMP were 5.7 vs. 14 mm and 8.26 vs. 16.08 mm for C1 and C2, respectively. This plot shows the errors for all external test datasets from both centers (combining the two datasets), wherein a significant difference between LCAP and LCMP and their absolute values was observed (*p* value < 0.01).

The error in DL performance was significantly larger for patients with a larger body habitus (*p* value < 0.05). In addition, the error was more considerable in females than males, but this difference was not statistically significant (*p* value = 0.25).

**Fig. 4:**
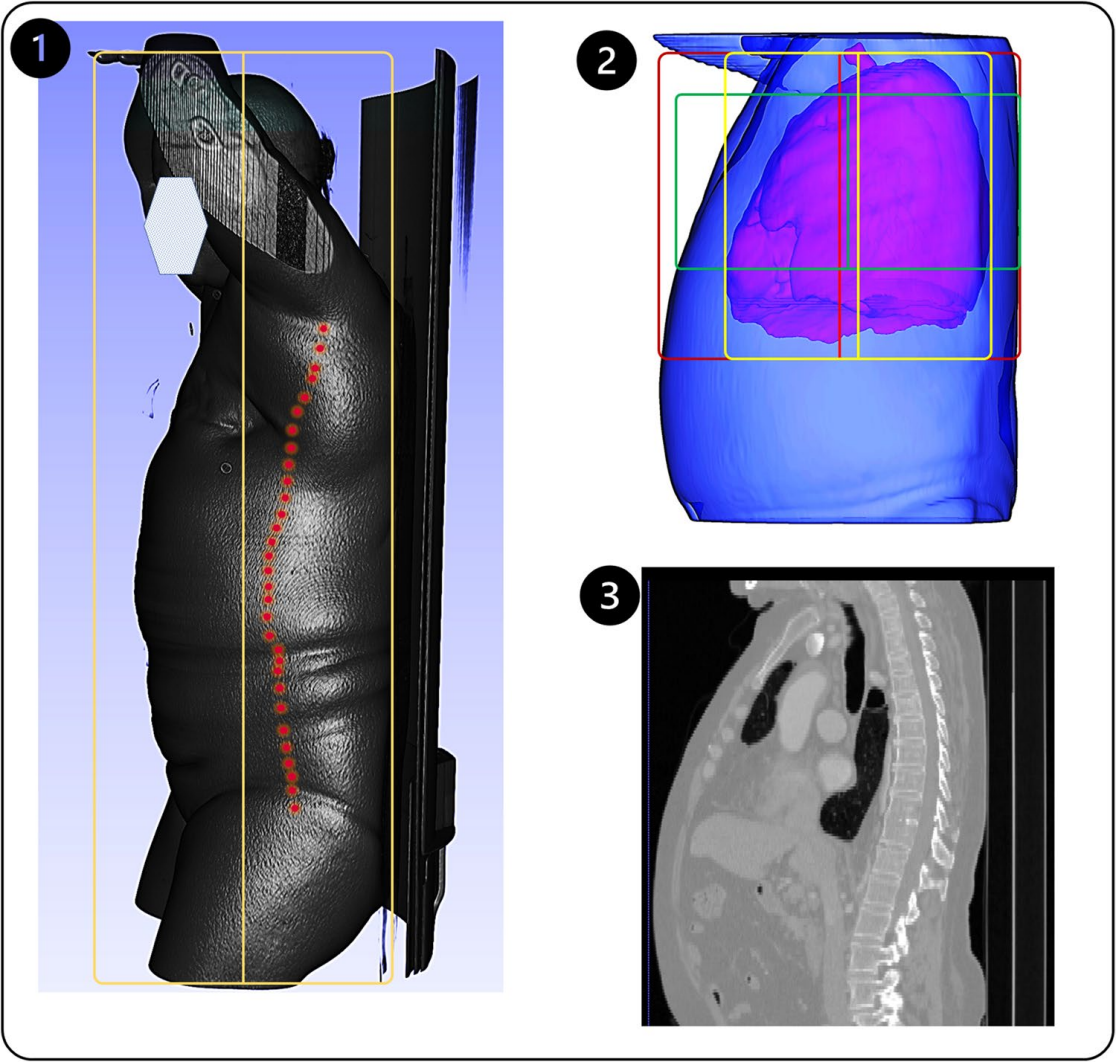
Effect of scan range on body centerline. (1) Lateral view of body contour (skin contour) from head to mid-thigh. The yellow box and line show the bounding box and the centerline can be detected by a visual camera with a zero-error accuracy. The red dots present the body centerline at each axial position. (2) A male patient chest 3D rendered image body contour (cyan) and lung segmentation (red). Three bounding boxes and centerlines are visualized. The red one shows body B.Box and centerline for chest CT typical scan range. The yellow one shows the lung B.Box and centerline for chest scan range. The green one shows the B.Box and centerline for typical cardiac scan range. (3) A coronal slice of the same patient shown in (2)

Figure 4 shows representative examples of axial and coronal slices of two patients. Figure 4(1) presents the total body centerlines, where the B.Box is highlighted in yellow, and the yellow line depicts the ideal B.Box. The centerline can be detected by a visual camera, while the red dots show the regional centerlines reflecting the body’s centerline for a limited axial range. Figure 4(2) shows the different centerlines for the body center and chest scan range (red), lung center and chest scan range (yellow), as well as the body center and cardiac scan range.

## Discussion

Patient mis-centering is a common issue in CT imaging, which could influence both patient radiation dose and image quality [31]. Our observations indicated that patient mis-centering is more than 10 mm in more than 60% of the cases in a clinical scenario. It should be mentioned that the table height was fixed in 78% of cases, demonstrating that the routine protocol was based on a fixed table height rather than a patient-specific table adjustment. In agreement with results reported in the literature, an average absolute BCMP (body centerline manual positioning) of $14.50\pm 10.40$ mm and $16.55\pm 9.72$ mm was obtained for C1 and C2, respectively. Akintayo et al [2] reported an average error of $14.7 \pm 17$ mm, whereas Dane et al [11] conveyed an average error of $16\pm 14$ mm. Moreover, Gang et al [12] recently reported a much higher mean error ($40.5\pm 24$ mm), which is comparable with the study of Sukupova et al [3], reporting $43$ mm average error. This observation could be related to the patient population, technologists’ skills/experience, and patient characteristics.

In this study, we developed a model to predict the 3D body contour from a single 2D localizer for chest CT scans. Consequently, we used these body contours to estimate the centerline of the body contour and its distance from the gantry isocenter, based on which automated table height adjustment was proposed to perform automatic patient positioning. In terms of accuracy, our method was comparable with studies that attempted to overcome the problem of mis-centering through a 3D camera either fixed on the ceiling in a single room or using portable cameras. Our errors in terms of absolute BCAP (C1$: 5.7\pm 5.26$ mm, and C2:$8.26\pm 6.96$ mm) are within the same range as reported in recent studies. Booij et al [9, 10] reported median and interquartile ranges of 5.4 and 6.4 mm for adults and 4.8 and 6.7 mm for pediatric patients, respectively, in positioning patients’ bodies in the gantry isocenter. In comparison, our results in terms of absolute BCAP median and interquartile range, considering the data from both scanners, were 5.25 mm and 6.94 mm, respectively. It should be noted that they excluded cases with an additional object on the patient’s body, such as a blanket, warm cloth, or fixation aid equipment.

Saltybaeva et al [13] reported an error of $7.0\pm 4.0$ mm in automatic body positioning on 68 chest CT cases using a visual camera, which is comparable with our results. However, they did not report cases with an absolute error of more than 20 mm, while we observed 45 cases with BCAP of more than 20 mm. Dane et al [11] developed a method that achieved an error of $6.8\pm 6.1$ mm, which is comparable with our results in terms of BCAP. It should be noted that they relied on a 3D camera and AP localizer and asked the technologists to confirm/verify the setup adjusted by the automatic model. Gang et al [12] scanned 127 COVID-19 patients twice with automatic and manual positioning and reported a $15.6\pm 8.3$ mm error in BCAP, which is noticeably higher than our results.

The significant merit of our proposed algorithm is that it does not require any additional device or time-consuming calibrations; besides, it is robust against the presence of additional objects, clothes, blankets, or tape fixators without any adverse effect on the positioning. It has been reported that the additional blanket on the patient’s body, blocking the vision of the 3D camera, is a common situation, especially in emergency and pediatric imaging. These external objects might lead to considerable errors in patient positioning, up to 70 mm [15].

Dane et al [11] emphasized the importance of the axial scan range in identifying the body centerline. Using a 3D camera and extracting body contour from the images, the whole body visible in the camera field of view is considered for distance and centerline detection. However, during spiral CT scanning, only a limited part of the body is scanned, e.g., the chest, abdomen, or brain. As shown in Fig. 4, the body centerline can vary significantly by changing the axial scan range, which limits the performance of cameras, even when the effect of clothes or blankets is ignored. The body centerline for chest imaging differs from the centerline for abdominal imaging; by the delimitation of the desired scan range on the localizer image, the centerline may change depending on the technologist’s decision, even in a typical chest scan [25, 32]. This fact can lead to mis-centering in the axial scan even with a complete and perfect performance of a 3D camera. Conversely, in our method, when the technologist selects the scan range on the localizer as routinely performed, the localizer is cropped, and the centerline fits exactly the spiral scan range indicated on the localizer image.

There is an intrinsic tradeoff in diagnostic radiology between image quality and radiation dose in terms of effective dose and organ-absorbed doses, such as the lens, breast, or gonads. Although simultaneous improvement in image quality and dose reduction can be achieved via positioning the patient at the isocenter, the image quality and radiation dose in specific organs can be affected in more complicated ways. Greffier et al reported that mis-centering affects the noise in the lung more than in soft tissue [6, 15]. The TCM system over/underestimates attenuation by changing the table height since higher table heights cause magnification in the AP localizer and increase tube current or tube potential (if the auto kVp option is available) and vice versa [7]. Besides, the shape of the bowtie filter increases the radiation flux in the gantry isocenter. It can be concluded that image quality and organ doses are affected by a combination of bowtie filter, TCM system estimation of attenuation, patient mis-centering, and organ position.

It should be noted that the BCAP was larger in female patients, though this was not statistically significant. The breast shape and position can significantly affect the body bounding box and centerline and might also adversely affect the performance of new dose reduction techniques that rely on tube current modulation [33]. Another critical point in identifying the body centerline could be considering or neglecting the breasts in centerline detection, which can change the calculated centerline. For patient positioning, the tradeoff between breast dose, internal organ doses, TCM performance, and image quality must be considered. Our results demonstrated a significant positive correlation between patient size and BCMP and BCAP, which is consistent the findings of Sukupova et al [3].

For specific indications, e.g., lumbar spine, cardiac or lung CT during the COVID-19 pandemic, image quality in a specific organ of interest is more important, whereas the target organ is suggested/preferred to be in the gantry isocenter [9]. We tested our method to distinguish the lung centerline from a localizer and its organ-based patient centering capability. The error in terms of LCAP was comparable with BCAP only for C1, which proves the capability of our method to perform organ-wise patient positioning. When comparing automatic and manual lung positioning (LCAP and LCMP), it should be noted that the technologists were not supposed to position the lung in the center of the gantry. In fact, lung positioning from the surficial anatomical marker is feasible neither by the technologist nor by the visual 3D camera.

With respiration, the body contour in the chest region changes. As such, positioning in a tidal breathing situation before starting the scan can cause some mismatches in body centerline detection [34], while the typical spiral chest CT breathing phase is end-inspiration. The localizer and the spiral acquisition can be in the same breathing phase through our proposed method to overcome this problem. This respiratory phase matching between the localizer and the spiral scan was one of the reasons for the good agreement reported in our results. One of our algorithm’s limitations is that it is scanner-specific; i.e., the trained network can only be implemented on the same vendor. This limitation is due to disparate pixel value definitions and pre-processing procedures adopted by the different vendors. We evaluated our method on two different CT scanners from two vendors and demonstrated that it outperforms humans and is comparable to alternative techniques using visual cameras. Transfer learning could be applied to address this issue in real clinical scenario by using a limited training dataset to implement the model in each center. However, alternative techniques relying on the use of a 3D camera are applicable only to a single room. Combining multiple methods of manual positioning and automated positioning using a 3D visual camera and our DL-based positioning might improve the accuracy, likely leading to eliminating the outlier cases. Each method has its limitations, and it would be interesting to check if combining the three models could circumvent the weaknesses of each one and improve the overall performance.

## Conclusion

We set out to overcome the problem of patient mis-centering by employing a deep neural network via generating a 3D composition of patients’ bodies from a single AP localizer image. The performance of the proposed network was comparable to other alternative techniques relying on 3D cameras. The advantage of the proposed approach is that it does not require any additional device; besides, it enables organ-based patient centering. This method could be implemented in clinical setting to aid technologists in diminishing the adverse effects of mis-centering on image quality and patient radiation dose.

## Abbreviations

AP: Anterior-posterior
BCAP: Body mis-centering during automatic positioning
BCGTH: Body centerline extracted from axial ground truth images
BCMP: Body mis-centering during manual positioning
CT: Computed tomography
CTDI: CT Dose Index
DL: Deep learning
LCAP: Lung mis-centering during automatic positioning
LCGTH: Lung centerline extracted from axial ground truth images
LCMP: Lung mis-centering during manual positioning
SNR: Signal-to-noise ratio
TCM: Tube current modulation

## Declarations

### Guarantor

The scientific guarantor of this publication is Habib Zaidi.

### Statistics and biometry

No complex statistical methods were necessary for this paper.

### Methodology

• retrospective

• experimental

• multicenter study
